# Flash Pyrolysis of Waste Tires in an Entrained Flow Reactor—An Experimental Study

**DOI:** 10.3390/polym16121746

**Published:** 2024-06-20

**Authors:** Balan Ramani, Arqam Anjum, Eddy Bramer, Wilma Dierkes, Anke Blume, Gerrit Brem

**Affiliations:** 1 Department of Thermal and Fluid Engineering, Faculty of Engineering Technology, University of Twente, 7500 AE Enschede, The Netherlands; 2 Department of Elastomer Technology and Engineering, Faculty of Engineering Technology, University of Twente, 7500 AE Enschede, The Netherlands

**Keywords:** tires, recycling, circular economy, sustainability, pyrolysis, entrained flow reactor, carbon black, oil, gas, mass and energy balances

## Abstract

In this study, a flash pyrolysis process is developed using an entrained flow reactor for recycling of waste tires. The flash pyrolysis system is tested for process stability and reproducibility of the products under similar operating conditions when operated continuously. The study is performed with two different feedstock materials, i.e., passenger car (PCT) and truck tire (TT) granulates, to understand the influence of feedstock on the yield and properties of the pyrolysis products. The different pyrolytic products i.e., pyrolytic carbon black (pCB), oil, and pyro-gas, are analyzed, and their key properties are discussed. The potential applications for the obtained pyrolytic products are discussed. Finally, a mass and energy balance analysis has been performed for the developed pyrolysis process. The study provides insight into the governing mechanisms of the flash pyrolysis process for waste tires, which is useful to optimize the process depending on the desired applications for the pyrolysis products, and also to scale up the pyrolysis process.

## 1. Introduction

A major challenge in the near future is to develop substitutes for raw materials and to recycle materials that are no longer available, scarce, or require a huge amount of fossil energy during production. One such material is the discarded waste tire, deemed unsuitable for reuse or retreading. Waste tires are predominantly landfilled, incinerated, or shredded to produce rubber granulates, each method presenting its own set of limitations. Tires are designed to operate under harsh environmental conditions and are therefore not biodegradable, causing them to stay in landfills for several years. Since tires are not compressible, the landfills require large landfilling volumes. The waste tire stockpiles are excellent breeding grounds for mosquitos and other insects that may spread diseases. Furthermore, landfills with waste tires pose a serious risk of fire. The incineration of waste tires for energy applications releases toxic combustion products, which when not treated properly can cause potential environmental pollution and serious health problems. The common recycling practices of waste tires as a raw material by producing rubber granulates and using them, for instance, in artificial grass fields, as antivibration solutions, or in rubberized asphalt, are often cascading in nature [1]. Even though this prevents the production of virgin material for these products, this is not a final solution. These products derived from waste tires will also have to be treated at their end of life, merely postponing the need for landfill or incineration.

In spite of the complexities that the disposal of waste tires brings, this also provides a valuable opportunity for resource conservation. The waste tires have great potential as a fuel or as a source from which valuable materials can be recovered. In order to valorize waste tires, different methods have been considered, one of which is the pyrolysis technology [2]. Pyrolysis has been acquiring renewed interest because of the advantages it provides to recycle complex waste materials. Pyrolysis is a thermal degradation technique of organic materials subjected to high temperatures in an inert atmosphere. In the case of pyrolysis of waste tires, the network of rubber polymers bonded to the carbon black surface gets decomposed at temperatures typically in the range of 500–800 °C, yielding oil and gaseous products [3]. The solid product remaining after pyrolysis predominantly comprises carbon black along with some inorganic filler and additive materials such as Si, Zn, Fe, etc. [4].

A majority of the research found in the literature on waste tire pyrolysis focuses on optimizing the yield and quality of the pyrolysis oil. Pyrolysis oil has a high heating value of up to 44 MJ/kg and has properties comparable to that of conventional fossil fuels [5], thereby finding application as an energy fuel, for instance, as a petroleum refinery feedstock or as a fuel in internal combustion engines [6,7,8], although a relatively high concentration of contaminants such as sulfur remains a barrier in this application, requiring a post-treatment technique like the desulfurization process in the case of sulfur removal [9]. On the other hand, pyrolysis oil also contains valuable chemical building blocks, such as benzene, toluene, xylene, limonene, etc., and optimizing the yields of these chemicals has lately been a subject of interest [10,11,12,13]. Hence, the oil derived from the pyrolysis of waste tires can find application either as a fuel source or can be used as a valuable feedstock for chemical industries.

Pyrolysis gas can be considered a valuable energy source due to its high calorific value, as it is mainly composed of hydrocarbons and hydrogen. The major application for pyrolytic gas is the combustion of a part of it to provide the energy required to operate the pyrolysis process, thereby making pyrolysis a self-sufficient process. Pyrolysis gas has a heating value in the range of 30–80 MJ/Nm^3^ and is a promising fuel when compared with natural gas, with a heating value of 35–40 MJ/Nm^3^ [14,15]. Even though combustion of pyrolysis gas as a fuel is promising, there are some environmental challenges that should be considered, mainly related to the emission of sulfur-based compounds [9].

Apart from the production of oil and an energy-rich gas, the thermal degradation of tires provides an opportunity to recover and reuse valuable chemicals present in them, such as carbon black. Carbon black is produced by the incomplete combustion of fossil resources, and it ranks as one of the top 50 industrial chemicals produced worldwide annually [16]. The main application of carbon black is its usage as a reinforcing filler in rubber applications, especially in the automotive industry, where it is responsible for the mechanical properties of the tire. Other industries in which carbon black finds application are printing inks, coatings, construction, and insulation, among others [17].

The annual global tire production was estimated to be around 2.2 billion units in 2017, which is roughly equivalent to 27 million tons (of which 5.1 million tons is produced in the EU [18]), and each year about the same weight of tires gets disposed as waste into the environment. This number keeps growing every year and is estimated to reach 2.7 billion units in 2022, equivalent to 33 million tons [19]. Depending on the type of the waste tire, approximately 20% to 27% of its weight consists of filler materials, mostly carbon black [20], and therefore around 6.5 to 9 million tons of carbon black can be recovered from these discarded tires every year. On the other hand, the worldwide production of carbon black through conventional processes using fossil resources is nearly 13 million tons annually, as estimated in 2015, which is further expected to increase to 19 million tons by 2022, worth EUR 17 billion [21]. The conventional carbon black production methods contribute approximately 5.7 kg of CO_2_ equivalent per kg of carbon black produced [5], estimated to equate to over 100 million tons of CO_2_ emission into the atmosphere every year. Carbon black recovery from pyrolysis of waste tires can thus constitute around 40% of the annual global carbon black requirement. It also offers a solution in clearing up two major environmental concerns, i.e., by reducing the amount of land-filled tires, and by decreasing the dependency on fossil resources and consequently reducing the CO_2_ pollution associated with conventional carbon black production by around 40%. This would generate a circular economy when the recovered carbon black is used to produce new sustainable tires.

Ash content in commercial carbon black is less than 0.5 wt. % [22], whereas in pyrolytic carbon black, ash content between 8.41 and 40.80 wt. % has been reported [5]. Ash present in pyrolytic carbon blacks mainly comprises silica and heavy metals such as zinc, iron, aluminum, calcium, potassium, etc., which do not evaporate at pyrolysis operating temperatures, thereby remaining as a solid fraction [5,23,24]. The heavy metals are added as activators and additives during the manufacturing process. They can also originate from the steel wires used in the tire as reinforcements that have not been completely separated from the tire feedstock before entering the pyrolysis process [25]. Ash can be removed from the pyrolytic carbon black by applying demineralization methods [23]. Ash content below 1 wt. % has been achieved using acid–base treatment techniques [24].

**Figure 1:**
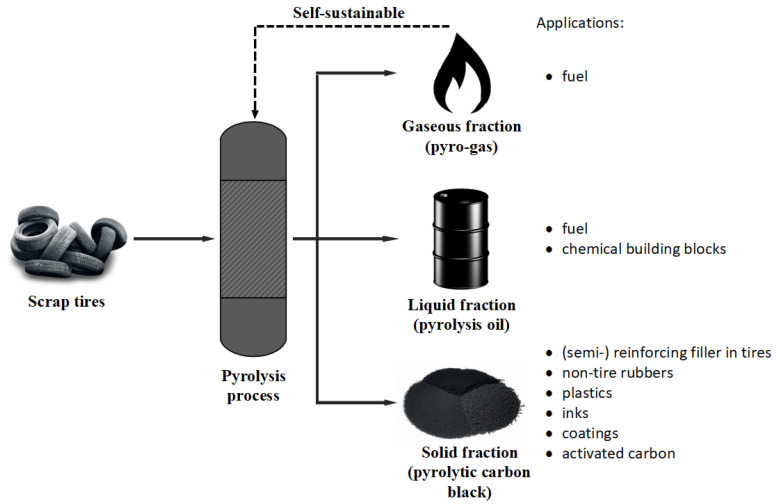
Schematic of the waste tire pyrolysis process with product applications.

Carbon black recovered from pyrolysis of waste tires that do not match the desired properties required for reuse in tire industries can find application in non-tire rubber, plastic, ink, and coating industries. Pyrolytic carbon black can also be upgraded to activated carbon using CO_2_ or steam activation, or cyclic oxidation techniques. Activated carbon produced from waste tires can be used for diverse applications, such as an adsorbent for water and gas purification, as a catalyst support, and so on [26]. A schematic overview of the waste tire pyrolysis process with potential applications for the different pyrolytic products is given in Figure 1.

Pyrolysis of waste tires is an extensively researched subject by several researchers using different reactor types and process conditions. Most commonly studied reactors for waste tire pyrolysis include fixed bed, auger, rotary kiln, stirred tank, fluidized bed, and conical spouted bed reactors [26]. Each of these reactors has its advantages and disadvantages, and the results vary greatly between the reactors. Depending on the application for the pyrolysis products, the optimal conditions for the selected reactor have to be found. Most of the research predominantly focused on optimizing the reactor conditions to obtain high-quality pyrolysis oil. Several review papers have been published since 2010, summarizing the results and findings of the waste tire pyrolysis research works [5,14,27,28]. The absence of a wide market for pyrolysis products, mainly for pyrolytic carbon black, has caused waste tire pyrolysis to not yet be industrially widespread [5].

In this study, an atmospheric downdraft entrained flow reactor is developed and tested for pyrolysis of waste tires. An entrained flow reactor is a fast pyrolysis reactor with a continuous mode of operation. One of the main advantages of the entrained flow reactor is the relatively short residence time of the feedstock particles inside the reactor. The reason to use a fast pyrolysis reactor is to reduce the occurrence of secondary cracking of the pyrolysis products by removing them from the pyrolysis zone within seconds, and thereby minimizing the growth of carbonaceous deposits on the surface of pyrolytic carbon black particles. Due to the short residence time of the particles inside the reactor, a smaller reactor volume is required as opposed to a slow pyrolysis process to obtain the same feedstock throughput. An entrained flow reactor for pyrolysis of waste tires is not much encountered in the literature, and an in-depth study is essential to understand its behavior with waste tires and its effects on the yield and properties of the pyrolysis products with a focus on the pyrolytic carbon black.

The purpose of this study is to examine the stability and product reproducibility of a continuous operation lab-scale pyrolysis process built using the entrained flow reactor. The process is tested for two different feedstock materials i.e., passenger car and truck tire granulates, to understand the behavior of the pyrolysis process for varied feedstocks. Reliable results on the yield and properties of the pyrolysis products are essential, which will provide valuable information to optimize the process to obtain the desired quality of products and to scale up the process to a large-scale plant.

## 2. Materials and Methods

### 2.1. Feedstock

**Figure 2:**
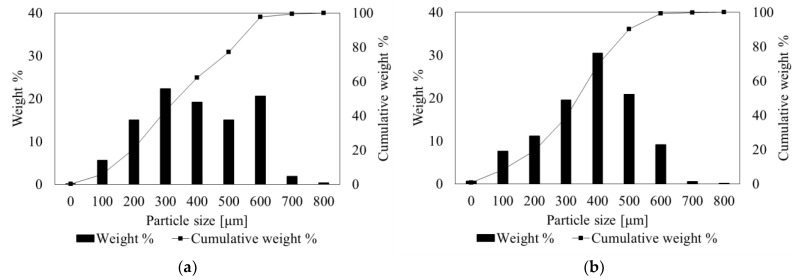
Particle size distribution of the feed tire particles: (**a**) passenger car tire; (**b**) truck tire.

The collection of waste tires involves gathering tires from various sources, including automotive repair shops, tire retailers, and municipal environmental centers. The waste tires are then sorted based on factors such as size, condition, and type. The tires that cannot be reused undergo mechanical shredding, a pretreatment process aimed at reducing them into smaller, uniform pieces or granulates of the desired size range. The aggregate size produced through mechanical shredding is crucial for efficient pyrolysis. It ensures uniform heating and processing of the tire material within the reactor, facilitating the conversion of waste tires into valuable products such as carbon black, oil, and gas. Two different feedstock materials have been used in this study: (1) passenger car tires (PCT) supplied by Granuflex, a Dutch waste tire recycling company, and (2) truck tires (TT) supplied by Continental Tires, a German tire production company. The raw materials are granulated with the particle size ranging from 0 to 800 µm, as seen in Figure 2 (corresponding to 20 US Mesh scale), with 95 wt. % of the distribution (D95) lying below the diameter of 700 µm for both PCT and TT materials, and free of steel cords and textile fibers.

### 2.2. Entrained Flow Reactor Setup

**Figure 3:**
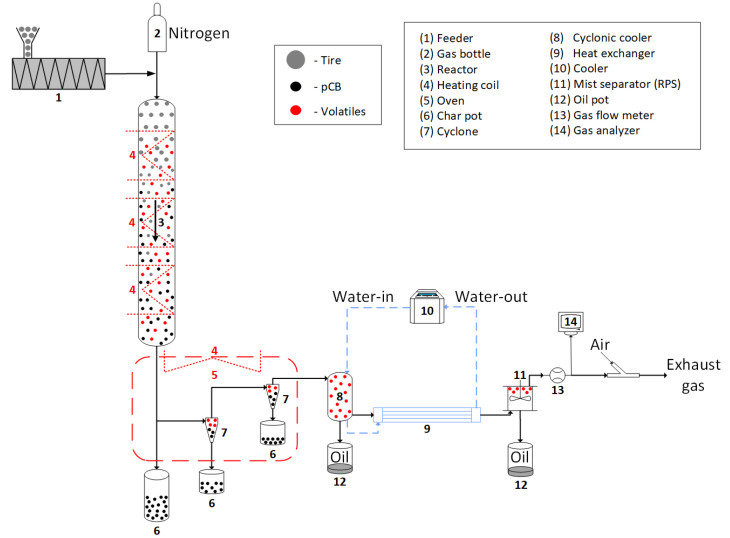
Schematic drawing of the entrained flow reactor experimental setup.

An atmospheric downdraft entrained flow reactor setup is used in this study to perform flash pyrolysis experiments on waste tires. Figure 3 shows a schematic of the reactor setup. The twin-screw feeding system feeds the rubber particles at a controlled rate of 1 kg/h into the reactor. The reactor is a stainless steel 316 cylindrical tube with a length of 4200 mm and an internal diameter of 50 mm. The reactor tube is divided into three heating zones electrically heated with high-temperature heating coils. The top zone is equipped with a 9 m heating coil with a power output of 2300 W. The central and bottom zones are each equipped with a 12 m heating coil with a power output of 2870 W. The temperature of each heating zone can be separately controlled by a K-type thermocouple mounted in the middle of the heating zone at the center point of the tube. The temperature can thus be accurately controlled, thereby having fewer temperature fluctuations along the tube length. Several other K-type thermocouples are installed along the length of the reactor to monitor the temperature profile inside the reactor. The feed particles are entrained in the nitrogen carrier gas flowing at a rate of 26 L per minute (with a gas-to-solid weight ratio of 2:1) through the reactor, during which the tire particles are pyrolyzed to form pyrolytic carbon black (pCB) and volatiles.

At the bottom of the reactor, the solid pCB product gets collected in a knockout char pot. Fine pCB particles can escape the knockout char pot, getting carried by the carrier gas along with the volatiles. These fine particles are separated from the gas flow using two cyclonic separators and trapped in the char pots placed at their bottom. The cyclonic separation system is placed inside an oven maintained at a temperature of 400 °C to prevent condensation of the pyrolytic volatiles. The volatiles leave the cyclonic separation system together with the carrier gas enter the quenching system, where they are cooled rapidly. Condensation of the volatiles take place in two stages. First, the hot gases pass through a cyclonic cooler, entering tangentially to create a swirling motion along the cold wall during which the heavy oil/tar gets condensed and collected at the bottom of the cyclonic cooler. The gases leaving the cyclonic cooler continue to pass through a 1 m-long jacketed cooling tube in which the remaining condensable gases are condensed to oil and enter a rotational particle separator (RPS), where the oil and any aerosol particles present in the gas get collected. The non-condensable gases pass through a flow meter to measure the flow rate. Then, the concentrations of different components present in the gas flow are measured using the gas analyzers before the gases leave to the exhaust.

### 2.3. Experimental Procedure

This work focuses on the development of waste tire pyrolysis process using an entrained flow reactor on a lab scale to provide reliable results that can be used for process scaling-up. All experiments are performed at 600 °C and atmospheric pressure using N_2_ as carrier gas at a flow rate of 1.6 Nm^3^/h. The mass flow rate of waste tires, both for passenger car and truck tire particles, is 1 ± 0.1 kg/h. The residence time of the feedstock inside the reactor is around 3–6 s and controlled by the flow rate of carrier gas. These variables are chosen so as to have a rubber conversion greater than 90 wt. %. The yields of solid and liquid products are measured directly by weight, while the gas yield is calculated by difference. After 30 min from the start of the experiment, the non-condensable gas samples are collected once the steady state is reached. Ten experiments are performed, each with passenger car and truck tire feedstock materials, respectively. The run time of each of the experiment is 1 h, with a total operation time of over 20 h without any technical problems.

### 2.4. Product Characterization

The feedstocks, referred to as passenger car tire and truck tire granulates were characterized by proximate analysis, ultimate analysis, X-ray fluorescence (XRF), heating value and density measurements. The solid fraction, also known as pyrolytic carbon black, was characterized by proximate analysis, toluene transmittance, ultimate analysis, X-ray fluorescence, scanning electron microscopy, pH value, heating value, surface area, porosity, and density measurements. The liquid fraction, known as the pyrolysis rubber oil, was characterized by ultimate analysis, water content by Karl Fischer titration, GCMS analysis, pH value, heating value, dynamic viscosity, and density measurements. The non-condensable gases, known as pyrolysis gases, were characterized using gas chromatography (GC).

The ultimate analysis was carried out in a ThermoFisher Scientific Flash 2000 Organic Element Analyzer and the XRF spectrum was taken with a Bruker S8 Tiger 4 kW wavelength dispersive XRF spectrometer. The proximate analysis was performed using a TA Instruments TGA 5500 thermogravimetric analyzer. The heating value was measured with an IKA C1 bomb calorimeter. Toluene transmittance was measured using an Agilent Cary 100 Bio UV-visible spectrophotometer. Scanning electron microscopy measurements were carried out in a Zeiss field-emission scanning electron microscope (FE-SEM). Surface area and porosity measurements were performed using a Gemini VII 2390 Surface Area Analyzer Micrometrics apparatus. pH values were measured using a Hanna Edge benchtop pH meter. Karl Fischer titration for measuring the water content in the oil was performed in an Metrohm 787KF Titrino titrator. Gas chromatography was performed using an Agilent 490 Micro Gas Chromatograph equipped with TCD/FID detectors and three channels: Molsieve 5 Å channel to analyze O_2_, N_2_, CO, CO_2_, CoX channel to analyze H_2_, and light hydrocarbon analyzer channel to measure the range of low-boiling hydrocarbons C_1_ to C_4_ in gaseous matrices.

## 3. Results and Discussion

### 3.1. Process Stability

**Figure 4:**
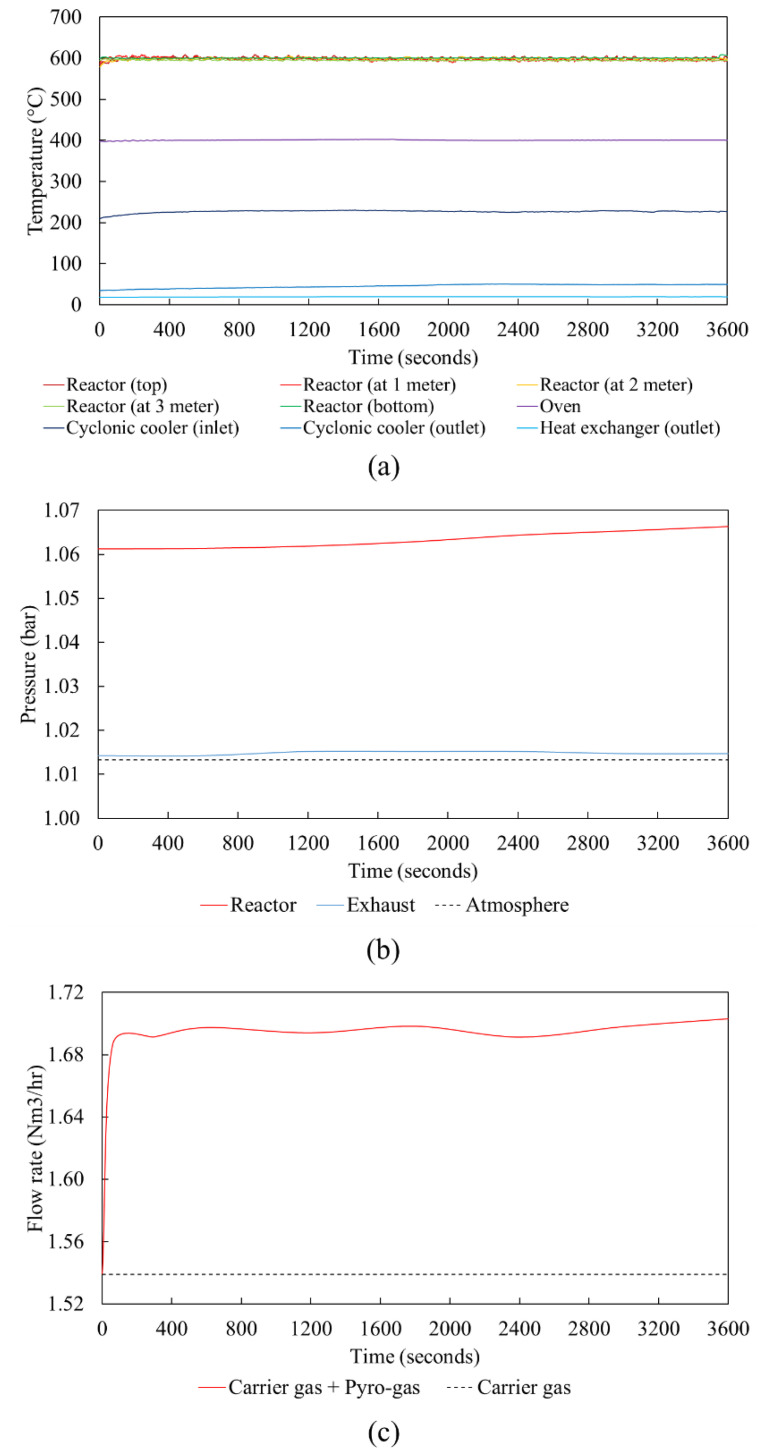
(**a**) Temperature profiles at different positions in the entrained flow pyrolysis setup, (**b**) pressure profiles at reactor inlet and at the exhaust of the setup, and (**c**) gas flow rate profile during a continuous pyrolysis production run.

Temperature, pressure, and gas flow rate profiles during a production run of 1 h of the waste tire pyrolysis process using the entrained flow reactor are shown in Figure 4. It can be seen from Figure 4a that the temperature profile is steady with fluctuations smaller than ±10 °C throughout the run time, showing excellent stability of the pyrolysis process. The reactor is maintained at 600 °C along the entire length of the reactor, with continuous temperature monitoring using multiple thermocouples placed every 1 m along the reactor length. The oven is maintained at a steady temperature of 400 °C to prevent condensation of the volatiles. The inlet temperature of the volatiles entering the cyclonic cooler is around 230 °C, which gets quenched down to 50 °C in the cyclonic cooler where the heavy oil/tar gets condensed. This is followed by a second stage of quenching in the tube heat exchanger to 20 °C, where the remaining condensable volatiles are condensed to form oil, leaving the non-condensable gases to exit together with the carrier gas. Figure 4b shows a slight over-pressure maintained to prevent leakage of atmospheric air into the pyrolysis system. The pressure profile in the reactor is almost steady over the run time, indicating no significant accumulation of the pyrolysis oil in the quenching system with continuous draining of the oil into the collection pots, thereby allowing continuous operation of the pyrolysis setup for a longer time period. The gas flow rate profile as seen in Figure 4c, shows that the carrier gas flow rate is maintained constant at around 1.54 Nm^3^/h throughout the experimental run time, with a steady production of pyrolysis gas at a rate of 0.16 Nm^3^/h.

### 3.2. Waste Tire

**Figure 5:**
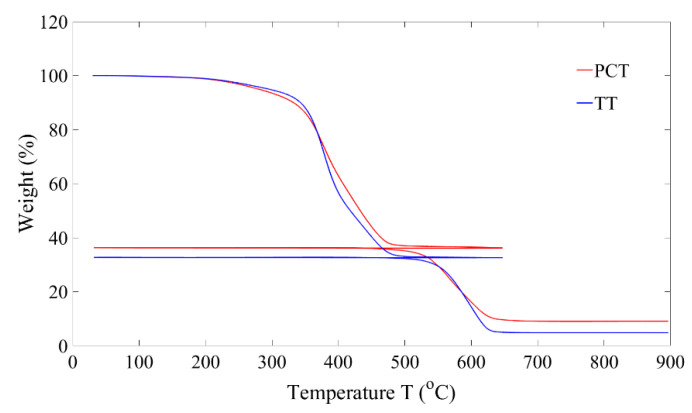
Thermogravimetric analysis of waste tires used: PCT; TT.

Proximate analysis, ultimate analysis, X-ray fluorescence, heating value, density, and particle size of the two different feedstock materials, i.e., passenger car tire (PCT) and truck tire (TT) granulates, are reported in Table 1. In the thermogravimetric analyzer (TGA), the feed material is heated in an argon atmosphere from 25 °C to 650 °C at a heating rate of 20 °C/min, during which the material starts to decompose, resulting in weight loss. At 105 °C, the measured weight loss corresponds to the moisture content present in the sample: at 650 °C, the weight loss corresponds to the amount of volatiles present in the sample. The sample is cooled to 25 °C, and the atmosphere is switched to air and heated to 900 °C at 20 °C/min. The sample is combusted, with the weight loss at 900 °C representing the fixed carbon content and the remaining sample weight corresponding to the amount of ash present. The weight loss trends with respect to temperature for both the feedstock materials are shown in Figure 5. Volatile content in proximate analysis corresponds to the oil, natural and synthetic rubbers present in the tire material, while the fixed carbon content represents the amount of carbon black present. Ash content corresponds mainly to the additives added during the rubber compounding process, with PCT having a higher ash content than that of TT, as seen in Table 1.

In the ultimate analysis, hydrogen content mainly corresponds to the hydrogen present in the polymer chain of hydrocarbons, as carbon black and ash do not have any significant amount of hydrogen present in them. Hence, for the pyrolytic carbon black, the hydrogen content can be a good indicator of the remaining amount of volatile impurities present on the carbon black surface. Carbon content can correspond to both carbon black and rubber polymers. Knowing that carbon black predominantly consists of more than 95% pure carbon with minimal quantities of oxygen, hydrogen, and nitrogen, the proportion of carbon content between the carbon black/polymer can be approximately estimated as 34/66% for both PCT and TT materials, respectively [29,30]. The rest fraction that cannot be measured using the ultimate analysis was estimated using X-ray fluorescence and was found to mainly be composed of silicon, zinc, sulfur, and oxygen, and trace amounts of other metallic elements. The heating value of both PCT and TT materials are notable i.e., 36.5 MJ/kg and 38.2 MJ/kg, respectively. The PCT material has a slightly lower heating value than the TT material due to its higher ash content and lower volatile content.

**Figure 6:**
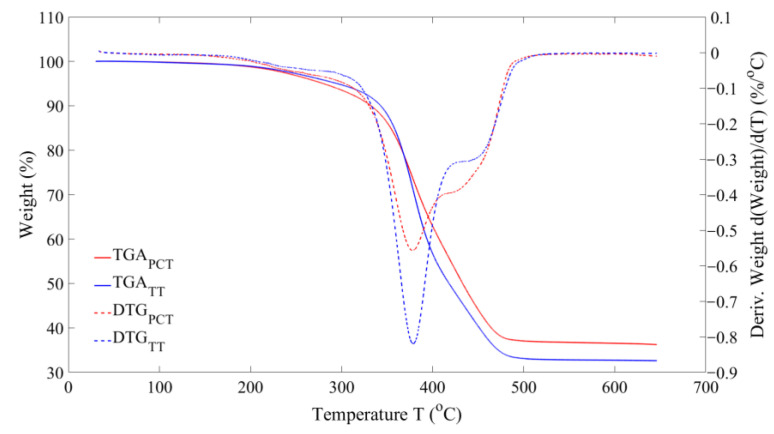
DTG/TGA curves of volatiles: PCT; TT.

Figure 6 shows the derivative thermogravimetric (DTG) plots of volatile decomposition for the two feedstock materials. The decomposition starts at around 200 °C and ends at 500 °C approximately with three different zones. The first zone represents the decomposition of oil additives in a temperature range of 200–250 °C, the second zone represents the natural rubber decomposition in a temperature range of 300–450 °C, followed by the third peak showing the synthetic rubber decomposition in a temperature range of 450–500 °C [5,31,32]. Hence, a minimum of 500 °C is required for complete decomposition of the rubber compounds. The second peak for TT is greater than that of PCT, while the third peak is greater for PCT than TT, showing the higher proportion of natural-to-synthetic rubber in TT than the PCT material.

### 3.3. Product Yields

**Figure 7:**
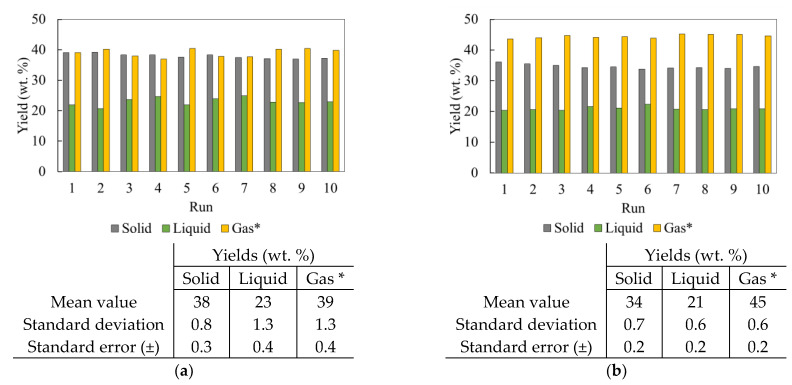
Product yields for two different feedstocks: (**a**) PCT; (**b**) TT. * Measured by difference, i.e., gas = 100—solid—liquid (in wt. %).

The product yields for the experiments performed using the entrained flow reactor for two different feedstock materials are shown in Figure 7. The results show that the process is very stable, with good reproducibility of pyrolysis products considering the complexities associated with such a continuous lab-scale production process. The yield of solid should be the sum of ash and carbon black present in the feed material i.e., 37 wt. % for PCT and 33 wt. % for TT. The additional weight is attributed to the volatiles remaining, which is around 1 wt. % for both the feedstock materials used, indicating a good conversion of rubber and removal from the solid product in the form of volatiles. The solid product from PCT has a higher yield compared to that from TT due to the higher quantity of ash present in the PCT material. The liquid-to-gas yields for PCT and TT materials are 23:39 wt. % and 21:45 wt. %, respectively. The higher gas-to-liquid yield ratio is because of the thermal cracking of volatiles into permanent gases due to their long residence time in the reactor. The gas-to-liquid yield from the pyrolysis process using TT feedstock is higher than that from the PCT feedstock. This is due to the higher proportion of natural-to-synthetic rubber in TT feedstock, as explained in the above section. The natural rubber starts to decompose at a lower temperature range than the synthetic rubber, resulting in a longer residence time for the produced volatiles in the reactor and leading to its breakdown into lighter permanent gases.

### 3.4. Solid Fraction

Proximate analysis, ultimate analysis, toluene transmittance, and heating value measurements are performed to check the reproducibility of pyrolytic carbon blacks obtained from different production runs in the same process conditions, and the results are summarized in Table 2 for passenger car and truck tire feed materials. The properties of pyrolytic carbon black depend significantly on the reactor type, feedstock composition, and process conditions, and good-quality pCB is crucial to make the waste tire pyrolysis process economically feasible, as it is one of the major components produced from the process. As mentioned in the previous section, the solid fraction obtained from a tire pyrolysis process is expected to predominantly consist of virgin carbon black, inorganics added during the tire production process and traces of unconverted rubber, and/or any condensed volatiles. The properties of pyrolytic carbon blacks show minimum variation across different production runs, as seen from Table 2 for PCT and TT feedstock, highlighting the stability of the entrained flow pyrolysis setup used in the study.

Over 90 wt. % of rubber content present on the carbon black surface is removed during the pyrolysis process, as observed from the reduction in volatile content from 63 to 5.6 wt. % for PCT feedstock and from 67 to 6 wt. % for TT feedstock. This claim is further supported by the simultaneous decrease in elemental hydrogen content from 7.3 to 0.7 wt. % and 7.8 to 0.9 wt. %, respectively, for PCT and TT feedstocks. This results in an increase in fixed carbon content, i.e., the carbon black present in solid product measured using the proximate analysis, from 28 wt. % to 71 and 79 wt. %, respectively, for PCT and TT feedstocks. The elemental carbon content measured in the ultimate analysis are 74 and 80 wt. %, respectively, for PCT and TT materials. The difference between the elemental carbon measurement from ultimate analysis and the fixed carbon measurement from proximate analysis represents the unconverted rubber or condensed volatiles remaining on the carbon black surface. The ash contents present in the pyrolytic carbon black are 23 wt. % for PCT and 15 wt. % for TT feedstocks. The higher ash content in the pCB obtained from PCT feedstock results from the larger quantity of ash present in the PCT feedstock than the TT feedstock. The elemental composition of rest fraction, which was not able to be measured using the ultimate analysis, was found using the X-ray fluorescence technique, which will be discussed in the following section.

Toluene transmittance is an ASTM method (D1618) for the determination of the light transmittance of a toluene extract from carbon black for use in the rubber industry using a spectrophotometer to measure the transmittance of UV light at 425 nm through this toluene extract [33]. The light transmittance value provides an estimate of the degree of discoloration caused by the toluene-extractable matter present on the surface of the carbon black. This method might not be applicable to carbon blacks with a high extractable-matter content. The transmittance values for pCBs obtained over different production runs are consistent with mean values of 26% and 23%, respectively, for PCT and TT feedstocks, showing good reproducibility of the product under similar process conditions using the pyrolysis setup.

Finally, the higher heating values (HHVs) of pCBs produced from different production runs are measured using the IKA C1 static jacket oxygen bomb calorimeter. The mean HHVs are 26.1 and 28.9 MJ/kg. respectively. for pCBs from PCT and TT feedstocks. with very small deviation between the samples from different production runs repeated under the same operating conditions. The lower heating value for the pCB from the PCT feedstock is due to its higher ash content than that from TT feedstock.

The reproducibility of the products under similar operating conditions is verified using the results discussed above, demonstrating the stability of the entrained downdraft flow pyrolysis setup used. Further characterization of the produced pyrolytic carbon black samples is discussed below.

X-ray fluorescence (XRF) is used for more complete elemental analysis on pyrolytic carbon black surfaces using a Bruker S8 Tiger 4 kW spectrometer. The rest fraction of pCB not measurable using the ultimate analysis, and so is measured using this method, and the results are summarized in Table 3. The sulfur content in pCB obtained from PCT and TT feedstocks are in the range of 2.1 and 1.8 wt. %, respectively, showing a retention of more than half of the sulfur in the solid fraction. Compounds such as ZnO and Fe_2_O_3_ react with sulfur to form ZnS and FeS, retaining the sulfur in the solid product. An increase in pyrolysis process temperature reduces the sulfur content in the solid and liquid phases [32]. The other elements, such as silicon and trace amounts of other metals, predominantly continue to remain in the solid fraction in the form of oxides.

The pH value of the filler material, i.e., carbon black, is an important property to be considered during the vulcanization of rubber compounds. When the pH value is below 7, there are acidic functional groups present on the carbon black surface that can consume the sulfur during the vulcanization process. This leads to an increased scorch safety time, which is not desirable, as the vulcanization process will take longer and hence there will be a loss in process efficiency. Furthermore, fewer cross-links may form, which can have a negative effect on the properties of the produced rubber compound. Hence, a higher pH value is desired for the carbon black, as it will increase the speed of the vulcanization process. Also, the mineral impurities on carbon black surface do not seem to alter its reinforcement properties, but by increasing the pH value of carbon black, they have a significant effect on the vulcanization speed [34]. The ASTM test method (D1512-15b) is used to indicate the pH value of the carbon black surface by measuring the pH of water in contact with the carbon black [35]. The pH values for the pyrolytic carbon blacks produced from PCT and TT feedstocks are both measured to be 7.8 ± 0.4 and 8.2 ± 0.2, respectively, and this will facilitate the vulcanization process.

**Figure 8:**
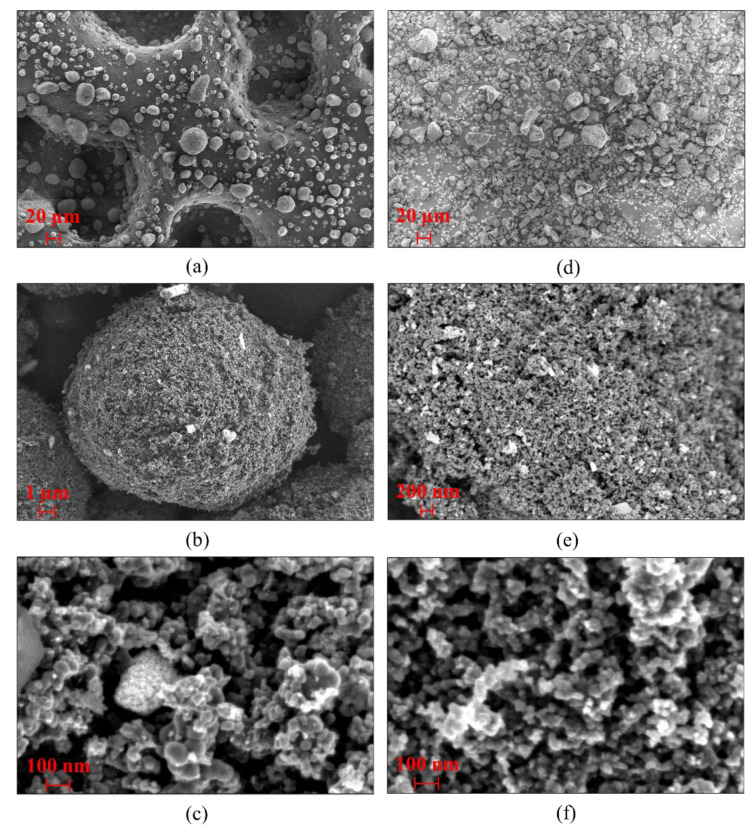
SEM images of pCB particles—PCT (**a**–**c**); TT (**d**–**f**).

A Zeiss FE-SEM (field-emission scanning electron microscope) with high-resolution imaging and superior material contrast is used to study the surface morphology of the pyrolytic carbon blacks produced from PCT and TT feedstocks, as presented in Figure 8. The high fraction of synthetic rubber in the PCT feedstock produces large voids and more channeling during degradation, causing low surface area and high porosity, while on the contrary, the high fraction of natural rubber in the TT feedstock produces small void spaces during degradation, causing more top surface area, as seen in Figure 8a and Figure 8d, respectively [32].

During the pyrolysis process, the inert constituents present in the feedstock material remain as ash on the surface of the solid product. The ash contents present on the surface of the pyrolytic carbon blacks produced from PCT and TT feedstocks can be seen as brighter particles contrasting on the darker carbon surface in Figure 8b and Figure 8e, respectively.

According to the International Carbon Black Association, carbon black exhibits a hierarchy of morphological features: primary particles, aggregates, and agglomerates. The primary particle is the fundamental building block of carbon black, strongly fused by covalent bonds into aggregates of colloidal dimension forming an aciniform (grape-like) morphology. Strong van der Waals forces maintain the integrity of the aggregate and promote the formation of agglomerates. While primary particle and aggregate sizes vary greatly depending on the carbon black grade and also within a given grade of carbon black, the primary particle size is essentially uniform within an individual aggregate. The size of the conceptual primary particle is in the range of 10–300 nanometers, with aggregates in the range of 85–500 nanometers, and agglomerates in the range of 1–100+ micrometers.

In a pyrolysis process, new carbon black is not created, but the range of virgin carbon black grades that were used in the tire production process, i.e., from N110 up to N772, are recovered. Hence, the pyrolytic carbon black will have the exclusive property of a mixture of carbon black grades present in the tire feedstock [27,36,37].

**Figure 9:**
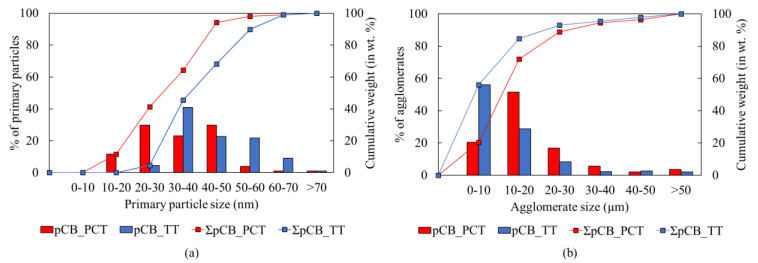
Particle size distribution for pCBs from PCT and TT feedstocks: (**a**) primary particles; (**b**) agglomerates.

The particle size distribution of the primary particles and that of the carbon black agglomerates measured from the SEM images in Figure 8 are plotted in Figure 9a and Figure 9b, respectively. Although grape-like clusters of the aggregates can be clearly visualized for the pCBs from both PCT and TT feedstocks in Figure 8c,f, it is difficult to distinguish and measure individual aggregate sizes from the SEM images. It can be seen from Figure 9a that the primary particles are distributed over a range of 10–70 nanometers, indicating the presence of a mixture of carbon black grades ranging from group number 1 to 7, as mentioned above [14,38]. The agglomerates as plotted in Figure 9b are in the range of 1 to 50 μm, with 95% of the distribution (D95) lying below a diameter of 30 μm for pCBs from both PCT and TT feedstocks.

The surface area of pCB is one of the most important quality indicators. The (external) surface area for a reinforcing filler material must be as large as possible in order to have a high contact area with the rubber. After the pCB samples are degassed at 125 °C for 24 h, the Brunauer–Emmet–Teller (BET) surface area is measured using a nitrogen adsorption isotherm at −196 °C in a Gemini VII 2390 Surface Area Analyzer Micrometrics apparatus. Using the physical adsorption of nitrogen gas molecules on the pCB surface, the BET surface area, i.e., the total specific surface area including the micropore surface area of the material, is measured. The results of the BET measurements for the pCBs produced from PCT and TT feedstocks are summarized in Table 4.

### 3.5. Liquid Fraction

The long-chain rubber compounds present in the feed tire material are decomposed into volatiles during the pyrolysis process. The condensable part of the volatiles is collected as pyrolysis oil, the properties of which are presented in Table 5. The H/C atomic ratios of the pyrolysis oil from PCT and TT feedstocks are around 1.1 and 1.2, respectively, meaning that the oil is primarily composed of aromatic compounds. A low O/C atomic ratio of 0.03 and low water content of around 1 wt. % for PCT- and 0.5 wt. % for TT-based pyrolysis oils result in high heating values of 40.7 ± 0.1 and 41.4 ± 0.1 MJ/kg, respectively, as measured in a bomb calorimeter. From the higher heating value (HHV) and elemental hydrogen content present in the oil, the lower heating value (LHV) is estimated to be 39.0 ± 0.06 and 39.7 ± 0.04 MJ/kg for PCT- and TT-based pyrolysis oils, taking into account the water content present in the oil. For the PCT and TT feedstocks, the sulfur content present in the oil is measured to be 1.7 ± 0.2 and 1.9 ± 0.04 wt. %, respectively, while the nitrogen content is found to be 1.3 ± 0.01 wt. %. A post-treatment process is therefore required for these pyrolysis oils derived from waste tires, as direct combustion of these oils will result in SO_x_, NO_x_ and N_2_O emissions. A pH value of around 8.0 ± 0.1 wt. %. for both the PCT- and TT-derived pyrolysis oils shows that these oils are almost neutral. The density of the oils at 20 °C is measured to be around 900 g/L, while the dynamic viscosities at 20 °C are found to be 16 ± 0.3 and 8 ± 0.3 cP, respectively, for the PCT- and TT-based pyrolysis oils. The higher viscosity of PCT-derived oil is attributed to its higher aromatic content compared to the TT-derived oil. It can be seen that the results show minimal variation between different test runs, showcasing excellent reproducibility also in terms of the properties of the oil produced using the developed entrained flow pyrolysis process.

### 3.6. Gaseous Fraction

The decomposition of waste tires produces a vapor containing H_2_, CO, CO_2_, H_2_S, and a high quantity of hydrocarbons such as C_1_–C_6_ [14,32,38]. The non-condensable part of the volatiles released during the pyrolysis process is collected as pyrolysis gas, and the volumetric concentration of the different gas components measured on a nitrogen gas-free basis are shown in Table 6. The pyrolysis gas properties show only small variations between the different production runs, again highlighting the very good reproducibility and stability of the entrained flow pyrolysis setup used in the study. The detected pyrolysis gas consists mainly of hydrogen, methane, ethane, ethene, propene, butene and butyne. The low CO and CO_2_ contents are associated with the low amount of oxygenated organic compounds present in the tire feedstock. The presence of high concentrations of C4 hydrocarbons can be traced to the depolymerization of butadiene (BR) and styrene–butadiene (SBR) rubbers present in the tire feedstock. The total detected fraction is around 80 vol. % for both the PCT- and TT-derived pyrolysis gases. The missing fraction is expected to consist mainly of C_4_+ hydrocarbons and hydrogen sulfides. The presence of C_4_+ hydrocarbons can be related to the depolymerization of styrene- and isoprene-based compounds, which are present in the synthetic and natural rubbers used in tire production, while the sulfides are from the decomposition of the sulfur links present in the vulcanized rubber structure [31,32,39].

The higher and lower heating values for PCT- and TT-derived pyrolysis gases are calculated for the detected gas compositions above. The pyrolysis gas can be used as a fuel gas due to its high heating value. which is even higher than that of the natural gas, with an HHV of 40.6 MJ/m^3^ and LHV of 36.6 MJ/m^3^ [15].

### 3.7. Mass and Energy Balances

The mass balance of the developed flash pyrolysis process from the passenger car tire feedstock study is considered using run 5. Table 7 shows the composition of the gas from the pyrolysis of PCT feedstock using the entrained flow reactor system, produced at a rate of 0.16 m^3^/h with an estimated density of 2.7 kg/m^3^. The total detected volumetric concentration of the pyrolysis gas is around 82%, while in weight concentration, it amounts to only 43%. The elemental weight concentration of the pyrolysis gas estimated by difference is C (85 wt. %), H (13 wt.), % S (2 wt. %), and N (0.2 wt. %), showing that over 98 wt. % of the pyrolysis gas is composed of hydrocarbons. Hence the missing fraction consists mainly of C_4_+ heavy hydrocarbons that were not measurable using the available gas chromatograph technique.

**Figure 10:**
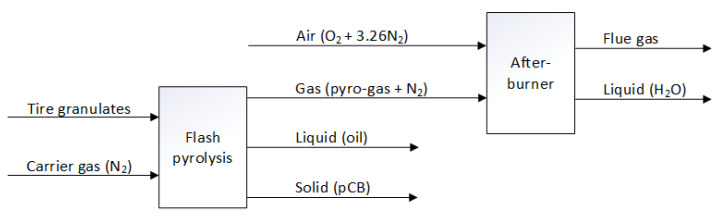
Schematic diagram of the afterburner analyzer.

In order to close the mass balance of the developed waste tire pyrolysis process, an afterburner combustion process is built and tested. The concept of the afterburner process is to combust and breakdown the heavy hydrocarbons present in the pyrolysis gases into lighter permanent gases that can subsequently be measured using a gas chromatograph. The afterburner is a combustion reactor made of a stainless steel tube with an internal diameter of 50 mm and a height of 800 mm, maintained at 1000 °C using heating coils that are regulated by a temperature controller. A distributor plate is located at the entrance of the reactor to ensure proper mixing of the pyrolysis gas with air for combustion. For a flow rate of 1.8 m^3^/h of inert nitrogen gas carrying the pyro-gas exiting the entrained flow pyrolysis process, an air flow rate of 1.8 m^3^/h is chosen empirically to ensure complete conversion of the heavy hydrocarbons present in the pyro-gas into lighter permanent gases. The residence time of the gases inside the combustion reactor is around 0.5 s. The combusted gases leaving the reactor are condensed to collect the water molecules (around 120 g/h) produced during the combustion of the hydrocarbons present in the pyrolysis gas. A schematic of the afterburner combustion process is shown in Figure 10.

After condensation, the volumetric composition of the remaining dry flue gas, with a flow rate of 3.6 m^3^/h, is measured using gas chromatography, and the results are summarized in Table 8. It is seen that the measured total volumetric concentration is close to 100%, indicating the complete breakdown of heavy hydrocarbons into lighter fractions. Besides the nitrogen gas, which is from the inert carrier gas used for pyrolysis and also from the air used for combustion, CO_2_ and CO are the main products of complete and incomplete combustion of the hydrocarbons present in the pyrolysis gas. H_2_ and CH_4_ are the remaining gases present in the flue gas, along with traces of lighter C2 hydrocarbons.

(1) $$tire+carrier\;gas\;(N_{2})+air\;(O_{2}+3.26N_{2})=pCB+oil+flue\;gas+H_{2}O_{\left(L\right)}$$

(2) tire=pCB+oil+pyrogas

(3) $$“pyrogas”+carrier\;gas\;\left(N_{2}\right)+air\;\left(O_{2}+3.26N_{2}\right)=flue\;gas+H_{2}O_{\left(L\right)}$$

The overall mass balance of the tire pyrolysis process with and without the afterburner combustion process is given by Equations (1) and (2), respectively, which can be reduced to Equation (3). Since the net “*pyrogas*” produced is the only unknown in Equation (3), substituting all the known values in this equation results in a pyrolysis gas yield of around 38 wt. %. This improves the overall mass balance of the tire pyrolysis process to 98%. The missing fraction of 2 wt. % that cannot be detected probably consists of sulfur- and nitrogen-based compounds in the pyrolysis gas.

The empirical formula of the different flow streams involved in the pyrolysis process calculated from the elemental analysis results are as follows: tire (C_207_H_224_O_8_X_4_S_2_N for PCT and C_255_H_284_O_7_X_3_SN for TT), pCB (C_338_H_39_O_38_X_18_S_4_N for PCT and C_308_H_40_O_22_X_10_S_3_N for TT), oil (C_78_H_89_O_2_SN for PCT and C_78_H_93_O_2_SN for TT) and for pyro-gas estimated by difference (C_134_H_253_S for PCT and C_391_H_704_S for TT). In the above empirical formulae, X represents the metallic elements present in the feedstocks, which gets retained predominantly in the solid pCB product, as seen from the pCB X-ray fluorescence results.

**Figure 11:**
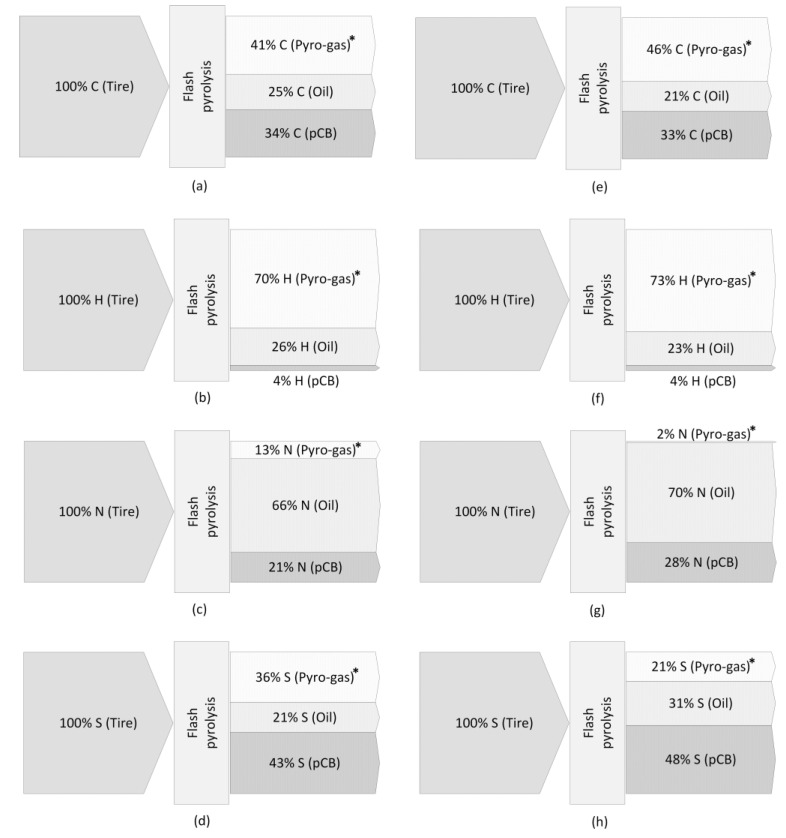
Elemental balances (C, H, N, S) of the flash pyrolysis process for PCT (**a**–**d**) and TT (**e**–**h**) feedstocks. * Measured by difference for the pyro-gas (i.e., pyro-gas = tire—pCB—oil) in wt. %.

An elemental balancing is transacted to understand the distribution of the elements present in the feedstock between the different fractions of pyrolysis products. The metallic elements in the feedstock are considered to stay in the solid pCB product, while the organic elements such as carbon, hydrogen, nitrogen, and sulfur are distributed between the different fractions after the pyrolysis process, as seen from the elemental analysis results. Figure 11 shows that nearly 50% of sulfur present in the feed was retained in the solid product, which is due to its reaction with the metallic elements present in the tire forming metallic sulfides that continue to remain in the solid product after the pyrolysis process. A predominant fraction of the nitrogen present in the feed is collected in the oil fraction after pyrolysis, while the remaining nitrogen in pCB is expected to be present in the functional groups on the carbon black surface. Most of the hydrogen present in the tire feedstock is from the rubber compounds, which are basically long chains of hydrocarbons deposited on the carbon black surface. During pyrolysis, the long hydrocarbon chains are broken down into smaller hydrocarbons that get removed in the form of volatiles, leaving a very low fraction of hydrogen in the pyrolytic carbon black product.

**Figure 12:**
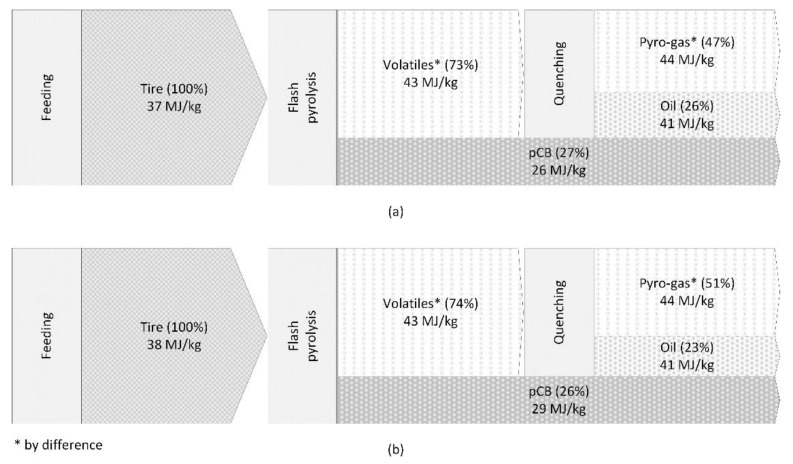
Energy balance of the flash pyrolysis process for (**a**) PCT and (**b**) TT feedstocks. * Measured by difference for the pyro-gas (i.e., pyro-gas = tire—pCB—oil).

A simplified energy balance of the waste tire pyrolysis process for PCT and TT feedstocks is summarized in Figure 12. The energy content of the different flow streams involved in the process—the energy content (HHV) of the feedstock (tire granulates: 100%), solid fraction (pCB: 27% of feed for PCT and 26% of feed for TT) and liquid fraction (oil: 26% of feed for PCT and 23% of feed for TT)—are measured using the bomb calorimeter, as explained in earlier sections, while that of the gaseous fraction (pyro-gas: 47% of feed for PCT and 51% of feed for TT) is calculated by difference. The energy required for heating and maintaining the reactor at the desired pyrolysis temperature, feeding tire granulates and inert carrier gas at the desired flow rates, quenching the volatiles produced during the pyrolysis process to separate between the liquid and gaseous fractions, and filtering out the aerosol particles trapped in the gaseous fraction is minimal and can be supplied by combusting part of the pyro-gas.

## 4. Conclusions

A novel flash pyrolysis system for waste tires has been developed in a downdraft entrained flow reactor. A series of tests has been carried out with two different feedstock materials i.e., passenger car tire and truck tire granulates. The process was proven to exhibit excellent stability, allowing continuous operation of the pyrolysis setup for a longer period of time, while retaining very good reproducibility of the pyrolysis products with minimal variations when operated under similar process conditions. An extensive characterization is executed to attain a detailed understanding of the different products, i.e., pyrolytic carbon black, oil, and pyro-gas, obtained from the flash pyrolysis process. This base knowledge provides insight into the dominant mechanisms of the process and will be helpful in further optimization of the process to modify the product yields and their properties, depending on the desired application for each of the pyrolysis products, by changing parameters like process temperature, residence time, particle size etc. Furthermore, a good closure of the mass and energy balances strengthen the reliability of the results achieved from the developed flash pyrolysis process, thereby providing valuable information for scaling up to a large-scale plant.

## Figures and Tables

**Table 1:** Properties of the waste tire materials.

	PCT	TT
Proximate Analysis (wt. %)		
Moisture	0.2 ± 0.01	0.2 ± 0.004
Volatiles	63.2 ± 0.3	66.9 ± 0.04
Fixed carbon	27.5 ± 0.5	27.8 ± 0.1
Ash	9.1 ± 0.7	5.1 ± 0.1
Ultimate Analysis (wt. %)		
C	80.4 ± 1	83.7 ± 0.2
H	7.3 ± 0.2	7.8 ± 0.1
N	0.5 ± 0.04	0.4 ± 0.03
Rest ^a^	11.8 ± 1	8.1 ± 0.3
X-ray Fluorescence (wt. %) ^a^		
Zn	3.2	2.4
Si	1.7	1.0
S	1.8	1.3
Fe	0.3	0.1
Ca	0.6	0.1
Na	0.1	0.1
K	0.1	0.1
Al	0.2	0.1
O	3.9	3.1
Heating value HHV (MJ/kg)	36.5 ± 0.2	38.2 ± 0.1
Particle size (µm)	0–800	0–800

^a^ Rest fraction measured using X-ray Fluorescence.

**Table 2:** pCB characterization—for passenger car tire and truck tire feedstocks.

Run	Proximate Analysis (wt. %)				Ultimate Analysis (wt. %)				Toluene Transmittance (%T)	HHV (MJ/kg)
	Moisture	Volatile	Fixed Carbon	Ash	C	H	N	Rest		
Passenger car tire feedstock										
1	0.3	6	71	23	73	1	0.3	26	27	26.3
2	0.3	7	71	22	74	1	0.3	25	29	26.2
3	0.3	5	71	23	74	0.8	0.3	25	26	25.9
4	0.3	5	72	22	73	0.7	0.2	26	27	26.0
5	0.4	6	71	23	73	0.6	0.3	26	25	26.1
6	0.3	6	72	23	74	0.7	0.3	25	28	26.1
7	0.4	5	72	22	73	0.6	0.2	26	24	26.0
8	0.4	5	71	23	75	0.6	0.2	24	26	26.1
9	0.4	5	72	23	74	0.6	0.2	25	25	26.0
10	0.4	6	72	23	75	0.6	0.2	25	27	26.0
Mean value	0.4	5.6	71	23	74	0.7	0.3	25	26	26.1
Standard deviation	0.05	0.5	0.4	0.4	0.7	0.1	0.04	0.7	1.5	0.1
Absolute error (±)	0.02	0.1	0.1	0.1	0.2	0.05	0.01	0.2	0.5	0.04
Truck tire feedstock										
1	0.5	8	77	14	81	1.1	0.4	18	22	29.2
2	0.5	8	77	14	80	1.1	0.4	18	21	29.1
3	0.5	6	79	15	80	0.9	0.3	18	22	29.0
4	0.6	6	79	15	80	0.8	0.3	19	26	28.9
5	0.6	5	79	15	81	0.9	0.2	18	22	28.4
6	0.8	5	80	15	79	0.7	0.3	20	22	28.6
7	0.5	5	79	16	80	0.8	0.3	19	24	28.9
8	0.9	6	79	14	81	0.8	0.3	18	25	29.3
9	0.6	5	78	16	79	0.9	0.2	20	20	28.3
10	0.7	6	79	15	81	0.7	0.3	18	21	29.0
Mean value	0.6	6.0	79	15	80	0.9	0.3	19	23	28.9
Standard deviation	0.1	1.2	1.0	0.6	0.7	0.1	0.06	0.8	1.8	0.3
Absolute error (±)	0.04	0.4	0.3	0.2	0.2	0.05	0.02	0.2	0.6	0.1

**Table 3:** XRF results of pCB rest fraction in wt. % for PCT; TT materials.

Elements	pCB_PCT	pCB_TT
Zn	4.5 ± 0.16	4.2 ± 0.07
Si	4.9 ± 0.21	3.2 ± 0.13
S	2.1 ± 0.06	1.8 ± 0.02
Fe	0.6 ± 0.04	0.1 ± 0.00
Ca	1.2 ± 0.03	0.2 ± 0.01
Na	0.6 ± 0.20	0.6 ± 0.04
K	0.1 ± 0.00	0.2 ± 0.01
Al	0.4 ± 0.00	0.1 ± 0.02
O + others	10.9 ± 0.50	7.5 ± 0.11

**Table 4:** BET measurements for pCBs from PCT and TT feedstocks.

	pCB_PCT	pCB_TT
BET surface area (m^2^/g)	58	87
Pore volume (cm^3^/g)	0.3	0.5
Pore size (Å)	232	236

**Table 5:** Oil characterization—for passenger car tire and truck tire feedstocks.

Run	Elemental Composition (wt. %)					H/C (-)	O/C (-)	Water Content (wt. %)	HHV (MJ/kg)	LHV (MJ/kg)	pH (-)	Viscosity at 20 °C (cP)	Density at 20 °C (kg/L)
	C	H	N	S	O + Others								
Passenger car tire feedstock													
1	86	7.6	1.3	1.9	3.3	1.1	0.03	1.0	40.8	39.3	7.7	14	0.889
2	86	7.2	1.3	1.5	3.7	1.0	0.03	1.1	40.3	38.9	7.9	16	0.904
3	87	8.6	1.3	n.m.	n.m.	1.2	n.m.	n.m.	40.9	39.2	8.1	16	0.891
4	86	8.5	1.3	n.m.	n.m.	1.2	n.m.	n.m.	40.6	38.9	8.3	16	0.913
5	87	8.6	1.3	n.m.	n.m.	1.2	n.m.	n.m.	41.0	39.2	7.9	16	0.855
6	86	8.3	1.3	n.m.	n.m.	1.2	n.m.	n.m.	40.7	39.0	8.2	16	0.932
7	87	8.2	1.3	n.m.	n.m.	1.1	n.m.	n.m.	40.4	38.8	8.4	17	0.861
8	86	8.6	1.3	n.m.	n.m.	1.2	n.m.	n.m.	40.8	39.1	8.0	14	0.918
9	87	8.8	1.3	n.m.	n.m.	1.2	n.m.	n.m.	41.1	39.3	7.9	14	0.951
10	86	8.3	1.4	n.m.	n.m.	1.2	n.m.	n.m.	40.5	38.8	8.3	15	0.828
Mean value	87	8.3	1.3	1.7	3.5	1.1	0.03	1	40.7	39.0	8.1	16	0.894
Standard deviation	0.4	0.5	0.04	0.3	0.3	0.07	0.0002	0.1	0.2	0.2	0.2	1.0	0.04
Absolute error (±)	0.1	0.2	0.01	0.2	0.2	0.02	0.0001	0.1	0.1	0.06	0.1	0.3	0.01
Truck tire feedstock													
1	86	8.0	1.3	1.8	3.0	1.1	0.03	0.6	41.4	39.7	7.5	8	0.907
2	86	8.2	1.3	1.9	2.2	1.1	0.02	0.4	41.2	39.6	7.7	9	0.936
3	88	8.9	1.3	n.m.	n.m.	1.2	n.m.	n.m.	41.7	39.9	8.1	7	0.947
4	86	8.7	1.2	n.m.	n.m.	1.2	n.m.	n.m.	41.5	39.8	8.0	8	0.885
5	85	8.9	1.3	n.m.	n.m.	1.3	n.m.	n.m.	41.7	39.9	8.2	7	0.948
6	86	8.5	1.3	n.m.	n.m.	1.2	n.m.	n.m.	41.4	39.7	7.8	9	0.941
7	87	8.5	1.3	n.m.	n.m.	1.2	n.m.	n.m.	41.3	39.5	7.6	9	0.923
8	86	8.4	1.3	n.m.	n.m.	1.2	n.m.	n.m.	41.4	39.7	8.4	7	0.986
9	85	8.8	1.3	n.m.	n.m.	1.2	n.m.	n.m.	41.5	39.7	8.2	7	0.962
10	87	8.6	1.2	n.m.	n.m.	1.2	n.m.	n.m.	41.5	39.8	7.7	7	0.955
Mean value	86	8.5	1.3	1.9	2.6	1.2	0.03	0.5	41.4	39.7	7.9	8	0.939
Standard deviation	0.8	0.3	0.04	0.1	0.6	0.04	0.003	0.2	0.2	0.1	0.3	0.9	0.03
Absolute error (±)	0.3	0.1	0.01	0.04	0.4	0.01	0.002	0.1	0.1	0.04	0.1	0.3	0.01

n.m.—not measured.

**Table 6:** Gas characterization—for passenger car tire and truck tire feedstocks.

Run	Gas Composition (vol%)												HHV (MJ/Nm^3^)	LHV (MJ/Nm^3^)
	H_2_	CO	CO_2_	CH_4_	C_2_H_6_	C_2_H_4_	C_3_H_8_	C_3_H_6_	C_3_H_4_	C_4_H_8_	C_4_H_6_	Total Detected		
Passenger car tire feedstock														
1	10.3	5.1	1.9	24.6	3.8	11.7	0.6	7.1	0.8	8.6	13.2	87.7	51.2	47.7
2	9.8	5.5	2.3	21.8	3.4	10.5	0.7	6.7	0.5	8.5	13.6	83.2	48.9	45.6
3	10.9	5.6	2.4	24.6	4.2	12.1	0.8	8.0	0.4	9.5	13.5	92.0	53.7	50.0
4	10.7	3.9	2.0	19.8	4.7	13.0	0.4	5.9	0.6	8.0	14.6	83.5	50.0	46.6
5	8.9	4.8	1.3	20.9	3.6	10.9	0.7	6.8	0.8	7.4	15.9	82.0	50.3	47.0
6	9.5	5.4	1.5	18.9	4.1	11.4	0.5	7.7	0.3	8.4	12.1	79.8	47.6	44.3
7	10.5	4.1	2.2	22.4	3.4	9.9	0.8	6.1	0.5	9.3	13.9	83.1	49.5	46.2
8	10.1	4.3	0.6	19.9	3.9	12.8	0.7	7.6	0.9	7.7	15.4	83.9	51.9	48.4
9	9.6	6.4	2.0	19.3	3.2	12.7	0.6	7.3	0.4	8.7	12.9	83.2	49.1	45.9
10	9.8	4.4	1.2	20.4	3.1	9.5	0.6	5.8	0.3	9.2	11.2	75.6	44.9	41.8
Mean value	10.0	4.9	1.7	21.3	3.7	11.4	0.6	6.9	0.5	8.5	13.6	83.4	49.7	46.3
Standard deviation	0.6	0.8	0.6	2.1	0.5	1.2	0.1	0.8	0.2	0.7	1.4	4.3	2.4	2.3
Absolute error (±)	0.2	0.3	0.2	0.7	0.2	0.4	0.04	0.2	0.1	0.2	0.4	1.4	0.8	0.7
Truck tire feedstock														
1	8.5	2.0	0.3	20.7	2.8	10.3	0.5	5.9	0.2	2.7	19.5	73.5	46.1	43.1
2	9.0	3.3	0.4	22.8	3.5	12.4	0.6	7.3	1.1	3.7	21.6	85.5	54.0	50.4
3	10.6	2.3	0.8	21.9	3.7	13.8	0.7	7.7	1.1	3.6	20.9	87.2	54.4	50.9
4	8.9	4.2	1.2	17.0	2.7	11.6	0.5	5.8	0.9	2.7	15.8	71.4	42.3	39.5
5	10.2	5.5	1.5	17.6	2.7	10.6	0.5	6.3	0.9	3.9	15.3	74.9	43.4	40.6
6	8.4	4.3	1.0	19.6	3.3	11.9	0.6	6.7	1.1	3.4	17.5	77.8	47.3	44.2
7	9.5	3.7	0.7	22.1	3.7	13.1	0.4	8.1	0.8	3.1	14.9	80.1	47.0	43.8
8	9.2	2.9	0.2	18.8	3.0	10.9	0.8	7.8	0.6	4.1	20.1	78.4	50.4	47.2
9	10.1	2.6	0.4	20.4	2.5	13.2	0.5	6.1	0.9	4.8	16.8	78.3	48.0	44.8
10	8.8	4.9	0.7	19.9	2.9	10.1	0.4	5.6	0.4	2.9	18.7	75.3	45.3	42.3
Mean value	9.3	3.6	0.7	20.1	3.1	11.8	0.6	6.7	0.8	3.5	18.1	78.3	47.8	44.7
Standard deviation	0.8	1.2	0.4	1.9	0.4	1.3	0.1	0.9	0.3	0.7	2.4	5.0	4.1	3.8
Absolute error (±)	0.2	0.4	0.1	0.6	0.1	0.4	0.04	0.3	0.1	0.2	0.8	1.6	1.3	1.2

**Table 7:** Pyrolysis gas composition in vol. % and wt. % for PCT feedstock (run 5).

	vol. %	wt. %
H_2_	8.9 ± 1	0.3 ± 0.1
CO	4.8 ± 0.5	2.2 ± 0.2
CO_2_	1.3 ± 0.2	1.0 ± 0.2
CH_4_	20.9 ± 1	5.6 ± 0.2
C_2_H_6_	3.6 ± 0.5	1.8 ± 0.2
C_2_H_4_	10.9 ± 1	5.1 ± 0.5
C_3_H_8_	0.7 ± 0.2	0.5 ± 0.1
C_3_H_6_	6.8 ± 1	4.8 ± 0.5
C_3_H_4_	0.8 ± 0.2	0.5 ± 0.1
C_4_H_8_	7.4 ± 1	6.9 ± 1
C_4_H_6_	15.9 ± 1	14.4 ± 1
Total detected	82.0 ± 4	43.0 ± 2
Missing fraction	18.0 ± 4	57.0 ± 2

**Table 8:** Volumetric composition of the flue gas exiting the afterburner.

	vol. %
H_2_	4.8 ± 1
CO	10.4 ± 1
CO_2_	3.4 ± 0.5
CH_4_	1.3 ± 0.5
C_2_H_6_	0
C_2_H_4_	0.1 ± 0.1
C_3_H_8_	0
C_3_H_6_	0
C_3_H_4_	0
C_4_H_8_	0
C_4_H_6_	0
N_2_	79.3 ± 2
O_2_	0.5 ± 0.2
Total measured	99.8 ± 2

## Data Availability

The original contributions presented in the study are included in the article, further inquiries can be directed to the corresponding author. This research is part of a publicly defended PhD thesis: https://doi.org/10.3990/1.9789036553834.
